# Lithium Point-of-Care Testing to Improve Adherence to Monitoring Guidelines and Quality of Maintenance Therapy: Protocol for a Randomised Feasibility Trial

**DOI:** 10.3390/ph18111683

**Published:** 2025-11-06

**Authors:** Jess Kerr-Gaffney, Priyanka Prakash, Victoria C. Wing, Allan H. Young, Oisín N. Kavanagh, John Hodsoll, Sarah Markham, David A. Cousins, Elliot Hampsey, Sameer Jauhar, David Taylor, Anthony J. Cleare, Rebecca Strawbridge

**Affiliations:** 1 Department of Psychological Medicine, Institute of Psychiatry, Psychology & Neuroscience, King’s College London, London SE5 8AB, UKbecci.strawbridge@kcl.ac.uk (R.S.); 2 Newcastle Magnetic Resonance Centre, Newcastle University, Health Innovation Neighbourhood, Newcastle upon Tyne NE4 5PL, UK; 3 Cumbria, Northumberland, Tyne and Wear NHS Foundation Trust, Newcastle upon Tyne NE3 3XT, UK; 4 Mental Health and Neurodegeneration and Dementia Theme, Translational and Clinical Research Institute, Newcastle University, Newcastle upon Tyne NE1 7RU, UK; 5 Division of Psychiatry, Imperial College London, London SW7 2AZ, UK; 6 School of Pharmacy, Newcastle University, Newcastle upon Tyne NE1 7RU, UK; oisin.kavanagh@newcastle.ac.uk; 7 Department of Biostatistics & Health Informatics, Institute of Psychiatry, Psychology & Neuroscience, King’s College London, London WC2R 2LS, UK; 8 Institute of Pharmaceutical Science, King’s College London, Franklin Wilkins Building, London SE1 9NH, UK

**Keywords:** lithium, protocol, randomised controlled trial, bipolar, depression, point of care

## Abstract

Lithium is the first-line treatment for bipolar disorders and a first-line augmentation option for treatment-resistant unipolar depression. Due to its narrow therapeutic window and risk of toxicity, people taking lithium require regular blood testing to monitor lithium levels in the body. However, studies have reported that only half of lithium-treated patients receive adequate lithium monitoring. This protocol describes a trial that will test the feasibility and acceptability of a point-of-care (POC) lithium blood testing programme in patients with unipolar or bipolar affective disorders taking lithium as a maintenance treatment. The primary objectives are to establish whether testing the effectiveness of POC testing is feasible, by assessing recruitment, attrition, and adherence to monitoring guidelines compared to participants randomised to testing as usual; to test whether the programme is acceptable to patients; and to measure potential contamination bias. The secondary objectives are to examine changes in health-related quality of life, the use of healthcare services, and depressive and manic symptoms to inform the design of a larger multi-site randomised controlled trial (RCT). This feasibility RCT will recruit 80 participants with affective disorders who are taking lithium. Participants will be 1:1 randomised to either POC monitoring or monitoring as usual where they will be followed up at three research visits over 30 weeks. The proportion of patients meeting guidelines for lithium monitoring will be examined, alongside measures of acceptability, wellbeing, and health economic data. POC testing has the potential to significantly improve patient safety and satisfaction with lithium treatment.

## 1. Introduction

Lithium, a mood stabiliser, is the recommended first-line long-term pharmacological treatment for bipolar disorder (BD) [1] and is used as an augmentation treatment for those with unipolar depression who have not responded to an initial course of antidepressants [2]. Due to its narrow therapeutic window and risk of toxicity, people taking lithium require regular blood testing to monitor lithium levels throughout the course of treatment. The National Institute for Health and Care Excellence (NICE) recommends that lithium concentrations should range between 0.6 and 0.8 mmol/L for those who are being prescribed lithium for bipolar disorder for the first time or up to 1.0 mmol/L for those who have previously relapsed or continue to experience symptoms whilst receiving lithium [1]. Those with lithium levels below the therapeutic window may be at increased risk of relapse, whilst those with levels above the therapeutic window are at increased risk of side effects and lithium toxicity [3]. This is problematic as lithium levels are highly variable and are influenced by the timing of the lithium dose and factors such as food and water intake, exercise, and concomitant medications [4,5].

NICE guidelines recommend that lithium levels be monitored every 3 months in the first year of treatment and every 6 months thereafter, although high-risk groups (e.g., older adults, those taking drugs which interact with lithium, or people at risk of impaired renal or thyroid function) should continue to be monitored every 3 months [1]. Levels should also be measured 1 week after any dose changes. However, audit data from NHS services within the UK reported that only 30% of patients met monitoring guidelines for the frequency of lithium testing in 2009 [6]. Following a national quality improvement programme [7], this rose to 48% [8]; however, half of patients continued to receive inadequate lithium monitoring. A later study in one NHS trust reported that only 50.7% of patients were within the NICE-recommended therapeutic lithium range, meaning that many patients may not be receiving effective symptom relief or are at risk of more severe side effects [9]. Studies outside of the UK have reported similar findings [10,11]. Various procedural and individual-level factors may contribute to poor monitoring rates, including a lack of communication between services and differing practitioner roles.

Currently, lithium testing is undertaken via laboratory testing, which typically involves patients attending a service specifically to have venous blood drawn by a nurse or phlebotomist, and the test results are then added to a patient’s health record within a few days. However, a number of inefficiencies have been identified in this system, including the need to book a separate blood test, with the appointment often being scheduled weeks after being ordered by the doctor; patients having to call practices on busy phone lines for their test results; non-clinical staff communicating test results; and a lack of formal protocols for communicating results [12,13]. The perceived burden of blood testing has been reported to contribute to the declining rates of lithium prescribing worldwide [14,15].

This study aims to test the feasibility of a novel, point-of-care (POC) lithium blood testing programme to improve monitoring in people with unipolar and bipolar affective disorder. POC testing allows for lithium serum levels to be measured by members of the patient’s treatment team during an appointment using finger prick testing, giving results in minutes. POC testing has revolutionised other areas of healthcare, for example, glucose monitoring in diabetes [16]. Recently, POC testing was introduced to one NHS trust for clozapine monitoring in schizophrenia. The new system was found to be highly acceptable to patients and staff: 90% of patients felt that the system improved clinical care, and staff felt that the system allowed for faster changes to prescription, the identification of non-compliance, and ease of administration [17]. The initiative received two (of six) findings of outstanding practice from the Care Quality Commission Inspection [18] and is now used routinely within several NHS trusts. Introducing POC testing into psychiatry may help bring mental health monitoring in line with physical health practices, such as those used in diabetes management.

The primary objective of this study is to test the feasibility and acceptability of POC lithium blood testing compared to usual monitoring for patients with affective disorders (bipolar disorder and unipolar depression) receiving lithium maintenance treatment, in a randomised controlled trial (RCT) design. Feasibility will be assessed via participant recruitment and retention rates, lithium discontinuation rates, and adherence to monitoring guidelines. Contamination bias (e.g., crossover effects) will also be assessed. Acceptability will be assessed via clinician and patient questionnaires. The secondary objectives are to measure health-related outcomes, service utilisation, mood, adverse events, and side effects.

## 2. Methods

### 2.1. Design

The Lithium Point-of-Care testing (LiPOC) trial is a multi-site, two-arm, rater-blinded RCT investigating POC lithium testing versus testing as usual (TAU) over 30 weeks. A total of 80 participants will be recruited across four sites in London and one site in the North of England: South London and Maudsley NHS Foundation Trust (SLaM), West London NHS Trust (WLNHS), South West London & St George’s NHS Trust (SWLSTG), Central and North West London NHS Trust (CNWL), and Cumbria, Northumberland, Tyne and Wear NHS Foundation Trust (CNTW). Participants will be randomised 1:1 to POC or TAU. Participants will remain under the care of their treating clinicians, meaning that their lithium testing will take place within their usual services, regardless of allocation.

### 2.2. Participants

Participants will be recruited from any service within the five participating NHS trusts. They may be identified via (a) direct research team communications with clinicians; (b) adverts within trust premises; (c) searches of the clinical records of patients who have consented to be contacted for research, such as SLaM’s Consent for Contact and linked Clinical Records Interactive Search; and (d) existing databases of patients who, through other routes (such as previous study participation), have consented to be contacted by researchers within the Centre for Affective Disorders, King’s College London, or CNTW.

Inclusion criteria are as follows: (a) aged 18 and above, (b) a diagnosis of a unipolar or bipolar affective disorder, (c) due to have monitoring for lithium treatment at the beginning of this study and three monthly for the upcoming 6-month period, and (d) be receiving care at one of the participating trusts. Exclusion criteria are as follows: (a) a planned discontinuation of lithium treatment during the 30-week study period; (b) an affective disorder diagnosis for which lithium is not licenced (i.e., schizoaffective disorder); (c) clinically significant manic symptoms, defined as a Young Mania Rating Scale (YMRS) [19] score ≥ 20; and (d) inability to provide informed consent.

We will also invite clinicians to participate in an optional add-on study at the end of their patient’s participation in the trial to assess clinician experiences with the intervention. The only inclusion criterion is that the clinician must have been involved in the POC intervention of their patient who was enrolled in our trial.

### 2.3. Procedure

**Figure 1:**
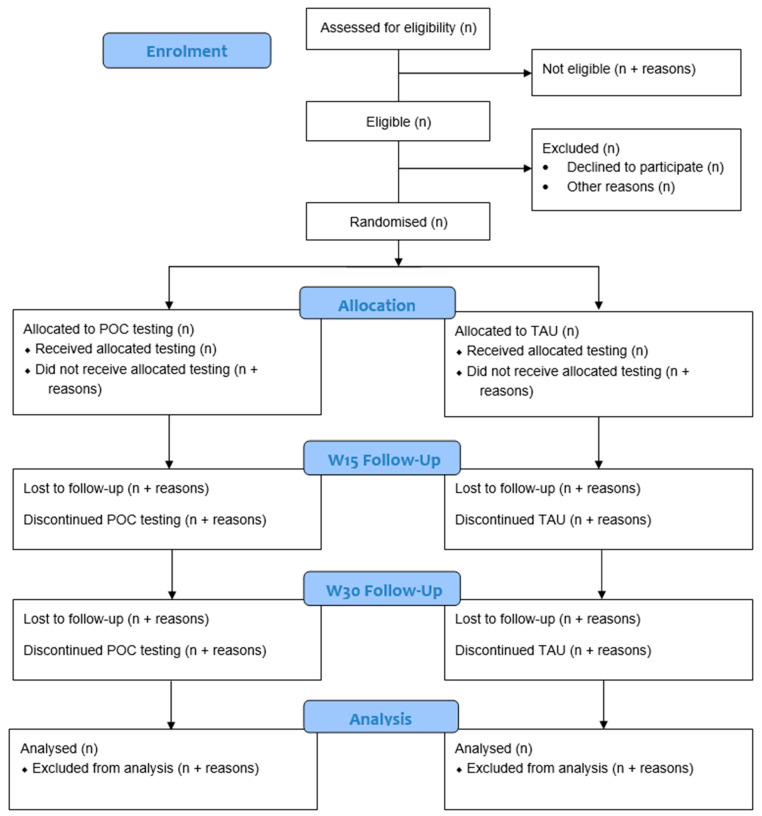
Study flowchart.

A study flowchart is shown in Figure 1. Participants will attend three visits: at week 0 (baseline), week 15, and week 30. All visits can take place remotely (i.e., via video call) or in person. Potential participants will first attend a screening visit to assess their eligibility and obtain verbal consent. Eligible participants will then be invited to attend a baseline visit, where written informed consent will be obtained, and demographic and baseline assessments will be administered (see Table 1). Following this, participants will be randomised, and the intervention will begin.

**Figure 2:**
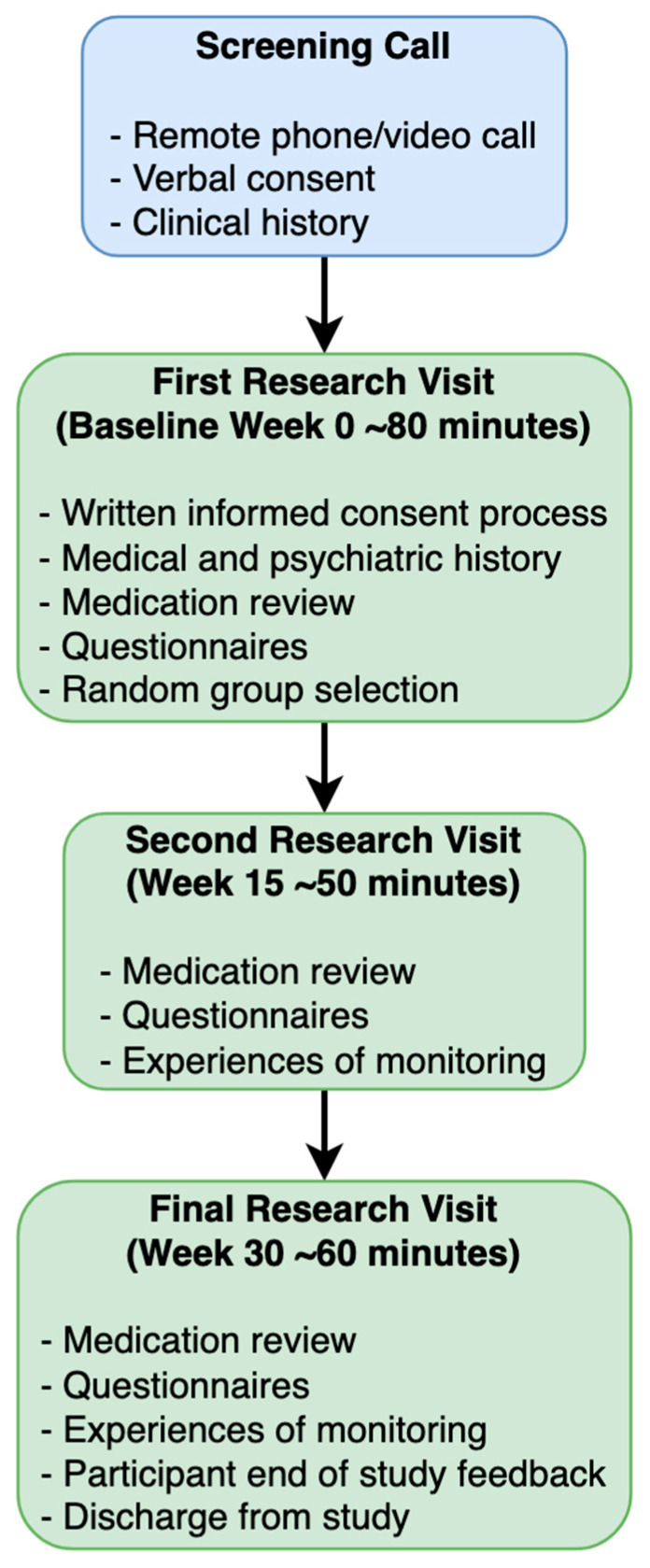
Summary of patient visits and assessments. Blue indicates screening (pre-study), green indicates baseline and follow-up period.

Follow-up visits will involve clinical and acceptability questionnaires, and any medication changes will be documented. In addition, researchers will obtain the levels of lithium, thyroid function test results, and serum levels of sodium, calcium, and creatinine via medical records. A timeline summarising patient visits during this study is shown in Figure 2.

### 2.4. Intervention

#### 2.4.1. POC Testing

Participants randomised to POC testing will have their lithium and creatinine serum levels tested with the Medimate device, a handheld tool which provides serum lithium and creatinine concentration from a finger prick blood sample within a few minutes. The device has been tested in line with international protocols, i.e., Clinical Laboratory Standards Institute Evaluation Protocols, which facilitate validation for manufacturers and laboratories in the US and EU. The device demonstrates an Allowable Total Error (ATE) rate for lithium levels below 10% or 0.1 mmol/L, exceeding both US CLIA regulations (maximum 20% or 0.3 mmol/L) and European consensus standards, and has a testing capacity of 0.2 mmol/L to 12 mmol/L [20]. It is also validated for measuring creatinine. Participants’ healthcare professionals will be asked to notify the study team when and where they are arranging the participants’ lithium test so that a study researcher can bring the testing device to administer the test to those in the POC arm. In this group, the Medimate device will be used for all lithium monitoring from randomisation up until week 30 of this study (provided agreement from clinicians and that they do not discontinue prior to this).

#### 2.4.2. TAU

Participants randomised to TAU will not receive any change to testing and will continue via laboratory testing as usual.

### 2.5. Randomisation and Blinding

Participants will be randomised 1:1 to POC or TAU within 24 h of completing the baseline assessment using King’s College Clinical Trials Unit independent web-based system. Randomisation will be stratified by site. The participant and healthcare professional will be notified of their allocation as soon as possible.

Participants and their clinicians will be aware of their treatment allocation. Researchers conducting the YMRS and Montgomery–Åsberg Depression Rating Scale (MADRS) outcome assessments will be blinded and will not have any other roles in the trial to maintain blinding. The trial statistician will also be blinded. The database will only refer to groups 01 and 02 without definition to maintain the blinding of the statistician.

### 2.6. Outcomes

Primary outcomes are as follows:

1. Feasibility
  - Participant recruitment rates over the trial period.
  - Participant retention and completion rates over 30 weeks.
  - Rates of lithium discontinuation over 30 weeks.
  - Adherence to lithium monitoring guidelines (proportion of participants meeting NICE guidelines for frequency testing and lithium levels within therapeutic range) over 30 weeks.
2. Patient acceptability
  - Patient monitoring acceptability questionnaires at week 15 and week 30.
  - Patient preferences for POC testing versus laboratory testing.
  - Perceived usefulness of POC monitoring.
3. Contamination bias
  - Monitoring rates between treatment-exposed versus non-exposed services.
  - Changes in monitoring rates from first to last patients recruited.
  - Participants’ monitoring rates from before trial (from electronic health records) versus during the trial.
  - Clinician survey about service changes during the trial.

Secondary outcomes are as follows:

1. Costs and economic outcomes
  - Health related-quality of life via the EuroQol-5D (EQ-5D) [21] at weeks 15 and 30.
  - Service utilisation via the Client Service Receipt Inventory (CSRI) [22] at weeks 15 and 30.
2. Mood and affective symptoms
  - Manic symptoms via the YMRS at weeks 15 and 30 [19].
  - Depression symptoms via the MADRS [23] at weeks 15 and 30.
  - Depression, mania, and anxiety symptoms via the Maudsley visual analogue scales (M3VAS) [24] at weeks 15 and 30.
3. Side effects and adverse events
  - Lithium side-effects rating scale (LiSERS) [25] at weeks 15 and 30.
  - Adverse events over 30 weeks.

### 2.7. Statistics

The sample size of 80 will enable the estimation of a 50% dropout rate with a confidence interval of ±10% [26].

Variables will be summarised using descriptive statistics (i.e., mean and standard deviation [SD] or median and interquartile range [IQR] or frequencies and proportions, as appropriate). Recruitment, retention, discontinuation, and measure completion rates will be described. The number and proportion of participants meeting NICE guidelines [1] for test frequency and therapeutic range compliance (usually 0.6 mmol/L–0.8 mmol/L but in certain specified circumstances either 0.8 mmol/L–1.0 mmol/L or 0.4–0.6 mmol/L) will be reported between study arms. Because our aim is to take three measurements per participant within the 30-week trial period, we are recruiting patients who require 3-monthly monitoring (with measurements at baseline/week 0, week 15, and week 30). However, due to the practical timing of clinic visits, some participants may only complete two measurements within this period; therefore two or three measurements will be considered adherent to monitoring guidelines, as long as they align with the participant’s scheduled monitoring timeframes. Acceptability questionnaires will be described quantitatively within subjects and between groups over time, as well as a qualitative analysis of narrative response questions.

The following will be analysed descriptively to investigate potential contamination bias (i.e., mean and SD or median and IQR or frequencies and proportions, as appropriate): (a) monitoring rates between treatment-exposed vs. non-exposed services, (b) changes in monitoring rates from the first to last patients recruited, (c) participants’ monitoring history from electronic health records, and (d) ratings from surveying clinicians about practice changes during the trial.

The unit costs of the intervention and use of other services will be estimated with the CSRI and compared between groups to identify the main cost impacts that should be measured in a future trial, in combination with health-related quality of life data from the EQ-5D to estimate quality-adjusted life years (QALYs). Generalised linear mixed models will estimate the between-arm differences at 15 and 30 weeks for the M3VAS, YMRS, MADRS, and LiSERS. These analyses will be used to estimate effect sizes for a future definitive RCT, as this feasibility RCT is not powered to detect treatment differences. The generalised linear mixed models will use all available data under a maximum likelihood framework. Missing follow-up data will not be imputed. Adverse events will be summarised descriptively between arms.

Analyses will be intention to treat (ITT). Sensitivity analyses may be carried out, excluding participants without clear information on their lithium treatment or monitoring.

Table 2 details key feasibility criteria to be met to proceed with a larger definitive RCT.

### 2.8. Trial Oversight

The trial received a favourable opinion from the London—South East Research Ethics Committee (25/LO/0381). This study will be conducted in compliance with the principles of the Declaration of Helsinki (1996) and Good Clinical Practice (GCP). King’s College London and South London and Maudsley NHS Foundation Trust are the sponsors of this trial. The trial was registered with the ISRCTN registry (ISRCTN17989376, on 27 May 2025). Participant recruitment commenced in September 2025 and will continue until September 2026.

A Trial Steering Committee (TSC) will provide oversight throughout the duration of the trial. The TSC aims to ensure the trial is being conducted to GCP and other regulatory standards, that participant welfare is being considered in the design and running of the trial, and that sufficient progress is being made with regard to key milestones (e.g., participant recruitment).

### 2.9. Data Security and Management

The chief investigator will act as custodian for the study data. All study data will be stored in line with the Data Protection Act and archived in line with sponsor requirements. The source data for the study will be collected on paper case report forms (CRFs). Data will then be input into the trial database, which will be hosted on a secure server hosted within King’s College London. Access to the trial database will be restricted through user-specific passwords to authorised research team members. The database will only store pseudonymised data, not including any direct patient identifiers (e.g., name, date of birth, or contact information). Unique participant identification numbers (PINs) will be used within the database and paper CRFs. Paper forms will be stored securely within locked cabinets at each site.

The study team will undertake reviews of the entered data where appropriate for the purpose of data cleaning and will request amendments as required. Following checks of data correctness and completeness, all data will be formally locked for analysis.

### 2.10. Patient and Public Involvement (PPI)

A lived experience advisory panel (LEAP) comprising individuals with experience of unipolar or bipolar depression and lithium treatment will meet throughout the trial, to provide insight into the real-world impact of the trial activities, processes, and findings. The trial team also includes a PPI lead, who will be part of the TMG and lead the LEAP and co-authored this protocol. Patient-facing documents were reviewed by the Feasibility and Acceptability Support Team for Researchers (FAST-R), a team of people with lived experience of mental health problems and their carers who advise on research proposals and documentation.

### 2.11. Dissemination

The results of the trial will be reported in peer-reviewed scientific journals and presented at conferences. A primary publication will include all primary and secondary outcomes listed in the protocol. Further publications may report exploratory analyses. The results will also be disseminated to the public via plain English summaries, with input from the LEAP.

## 3. Discussion

This protocol describes a feasibility RCT which aims to test the utility of a novel POC testing method for lithium monitoring in the UK. Data gathered on feasibility, acceptability, and adherence to monitoring guidelines will be used to inform a larger confirmatory trial, which would establish whether POC testing is clinically effective and cost-effective. If POC testing is found to be effective in improving lithium monitoring in those with unipolar and bipolar affective disorders, this could lead to several benefits for this population. If there is an improvement in the proportion of time that participants’ lithium levels are kept within the therapeutic range, this could improve affective symptoms and reduce the side effects of lithium, enhancing quality of life. Further, POC testing may lead to direct and indirect cost benefits, for example, by eliminating the need for a separate appointment for a blood test and therefore reducing service use requirements and patient time off work. Further, prior research using POC testing for those taking antipsychotics has indicated that patients felt more involved in their own care and that receiving the test results almost immediately helped them understand the purpose of testing [17]. This could lead to improved engagement, trust, and communication between patients and their healthcare team.

This trial has a number of strengths and limitations. The trial will use a novel drug level monitoring method that has been shown to be acceptable and preferred over laboratory testing in a similar patient group [17]. Further, although this is a feasibility trial, the inclusion of five NHS sites across two regions of England will help improve the generalisability of the results, ensuring that the results are not an artefact of processes at one NHS trust. Although double-blinding is not possible in this trial (patients and at least some of the patient’s healthcare team would need to know their allocation), blinded-outcome raters will complete assessments for key clinical outcomes with patients to reduce bias. Related to this, it is possible that clinicians who are notified that their patient is taking part in a lithium monitoring trial will take more care to follow clinical guidelines for lithium monitoring. Measures to reduce contamination bias will be taken; for example, when patients’ clinicians are notified of their involvement in the trial, minimal detail on the intervention will be given to clinicians whose patients are in the TAU arm to reduce the chance that they will change their monitoring practices. They are told that the patient is taking part in a trial relating to lithium treatment but not that the trial relates to lithium monitoring. Further, a multi-method assessment of contamination bias is a primary objective of this trial, incorporating analyses of monitoring rates from patients before they entered the trial, comparing monitoring rates from exposed versus non-exposed services, changes in monitoring rates across the recruitment period, and feedback from clinicians.

Because this is a feasibility trial, inferences about efficacy cannot be made from the results. A further limitation of this trial is that we are only including patients who require 3-monthly monitoring, therefore excluding a large proportion of patients on lithium maintenance treatment who require monitoring every 6 months. This decision was made in order to have an adequate number of scheduled monitoring appointments per participant within the time constraints of a feasibility trial. If this trial is successful, we would hope to also include those on 6-month monitoring in the larger confirmatory trial that would follow, which may include a longer follow-up period. A 12-month follow-up period would allow for the inclusion of patients who require 3-monthly or 6-monthly monitoring, with 4 or 2 expected monitoring appointments within the trial follow-up period, respectively. This would also allow longer-term adherence and efficacy to be assessed. However, a 12-month follow-up period presents challenges with regard to research investment. Finally, although the POC testing method provides lithium and creatinine serum levels, it does not provide monitoring of other parameters that the clinician may require or are recommended while taking lithium, such as urea and electrolytes, calcium, and thyroid function. NICE guidelines recommend that these parameters be monitored every 6 months or more frequently in certain circumstances [1].

The quality of lithium monitoring in the UK and elsewhere is currently poor, potentially contributing to poor health outcomes in those with unipolar and bipolar affective disorders. Known issues with current blood monitoring practices may lead to patients being outside the therapeutic range for lithium, putting them at risk of relapse or sub-optimal symptom management if levels are too low or compromising physical health if levels are too high. The LiPOC trial aims to build on previous work introducing POC testing to psychiatry, with the hope of improving care and quality of life in this population. Further, despite lithium being the most efficacious drug for preventing mood episodes in bipolar disorder, the rates of prescribing are declining [27]. If POC testing is eventually adopted in healthcare services and is perceived by clinicians and patients as less burdensome and more efficient than laboratory testing, clinicians may be more likely to prescribe lithium to their patients, providing more patients with the chance to trial an effective treatment for their condition.

## Figures and Tables

**Table 1:** Study procedures by visit.

Procedures/Measures	Screening (Pre-Study)	Baseline (W0)	Follow-Up 1 (W15)	Follow-Up 2 (W30)
Informed consent process	X	X		
Medical and psychiatric information	X	X		
Sociodemographic information	X	X		
Medication and lithium review	X	X	X	X
Randomisation		X		
EuroQol-5D (EQ-5D)		X	X	X
Client Service Receipt Inventory (CSRI)		X	X	X
Young Mania Rating Scale (YMRS)	X	X	X	X
Montgomery–Åsberg Depression Rating Scale (MADRS)		X	X	X
Maudsley visual analogue scales (M3VAS)		X	X	X
Lithium side-effects rating scale (LiSERS)		X	X	X
Patient monitoring acceptability questionnaire		X	X	X
Participant end of study feedback questions				X

**Table 2:** Go–no-go criteria to be met to proceed with an efficacy trial.

	Go—Proceed with RCT	Amend—Proceed with Changes	Stop—Do Not Proceed Unless Changes Are Possible
Participant recruitment	80 in 12 months	80 reached but in >12 months	53 not reached by 12 months
POC monitoring acceptability	≥80% rating POC as “acceptable”	30–79% rating POC as “acceptable”	<30% rating POC as “acceptable”
Participant attrition rate	≤20% attrition	21–50% attrition	>50% attrition
Adherence to lithium monitoring guidelines	>60% adherence	30–60% adherence	<30% adherence

## Data Availability

No new data were created or analyzed in this study. Data sharing is not applicable to this article.

## Abbreviations

The following abbreviations are used in this manuscript:

ATE	Allowable Total Error
BD	Bipolar Disorder
CNTW	Cumbria, Northumberland, Tyne and Wear NHS Foundation Trust
CNWL	Central and North West London NHS Trust
CSRI	Client Service Receipt Inventory
EQ-5D	EuroQol-5D
FAST-R	Feasibility and Acceptability Support Team for Researchers
GCP	Good Clinical Practice
ITT	Intention to Treat
IQR	Interquartile Range
LEAP	Lived Experience Advisory Panel
LiSERS	Lithium Side-Effects Rating Scale
M3VAS	Maudsley Visual Analogue Scales
MADRS	Montgomery–Åsberg Depression Rating Scale
mmol/L	Millimoles Per Litre
NHS	National Health Service
NICE	National Institute for Health and Care Excellence
POC	Point of Care
RCT	Randomised Controlled Trial
SD	Standard Deviation
SLaM	South London and Maudsley NHS Foundation Trust
SWLSTG	South West London & St George’s NHS Trust
TAU	Treatment As Usual
TSC	Trial Steering Committee
WLNHS	West London NHS Trust
YMRS	Young Mania Rating Scale
